# Some Nervous Troubles Associated with Diseases of the Ear*Translated from *L’ Union Médicale du Canada*, August, 1897, by Dr. L. B. Grandy, Atlanta.

**Published:** 1897-11

**Authors:** A. A. Foucher

**Affiliations:** Montreal, Canada


### SOME NERVOUS TROUBLES ASSOCIATED WITH DISEASES OF THE EAR.*

By PROFESSOR A. A. FOUCHER,

Montreal, Canada.

Nervous manifestations, occasioned by different maladies of the ear, often present themselves in practice with characters which mask their origin and which divert the physician from their true signification. Even the specialist, although familiar with ordinary nervous troubles which originate in the ear, may be led into error as to the exact nature of the symptoms which he observes, or may allow some to pass unnoticed, especially when there exist other lesions in the neighborhood of the auditory apparatus. Thus a cough, or a dysphagia, may easily find an explanation, and be attributed to some trouble in the superior or inferior respiratory passages, when the true origin is in the ear, with or without pain or deafness.

The rich nervous network which is distributed to the external and middle ear, and which contains filaments from the trigeminus, facial and glossopharyngeal, and sensory anastomoses of the pneumogastric, facial and sympathetic, explains sufficiently the exquisite sensitiveness of the ear and its prompt reaction to influences from without. This intimate interlacing, in such a small space, of the branches of these different pairs, renders the ear subject to reflexes of the most varied character. An irritation, however light, at any point in the external auditory canal, or of the drum membrane, or of the tympanum, may produce toothache, paroxysms of cough, asthma, salivation, uncontrollable vomiting, hemicrania, dysphagia, paralysis, epilepsy, chorea, hysteria, vertigo, and a number of other symptoms of psychic nature.

However, reflex nervous phenomena do not necessarily accompany these lesions about the ear. Politzer removed a piece of pencil, three centimeters long, which had remained in the auditory

*Translated from *L' Union Médicale du Canada*, August, 1897, by Dr. L. B. Grandy, Atlanta.

canal for fifty years, without causing more than a certain degree of deafness. Lucae, Zaufal and Rein have observed similar cases. On the other hand, there is a long list of very interesting cases which prove that irritation of this region may produce not only violent phenomena along the course of the trigeminus and vagus nerves, but also prolonged general nervous attacks which disappear only with the irritant agent which has caused them.

Arnold cured a young girl afflicted for a long time with a persistent cough and frequent vomiting by removing two beans from the external ear. Toynbee and Politzer stopped a stubborn cough by the extraction of a sequestrum of bone. Fabricius von Hilden cured a girl, the victim of epilepsy, of a dry cough, of anesthesia of the entire half of the body and of atrophy of the left arm, by removing a piece of glass introduced eight years before into the auditory canal. Gellé, Baratoux, Jackson, Noquet of Lille, Boucheron and others have cited cases of epilepsy originating in the ear, and being cured when the disease of the ear was treated. Moos has cured a hemiplegia caused by an insect in the canal. Bertrand (cited by Calmette) has cured a girl, five years old, who, in the course of a purulent otitis, fell to sleep in a field and was awakened by buzzing noises and some pain. The trouble increased up to the point of causing convulsions, followed by syncope. The patient's face became bloated, the eyes dull, nostrils dilated, the mouth open, from which issued a frothy saliva; respiration became labored and the pulse small, feeble and intermittent. All the left side of the head was of a livid color, swollen and threatened with gangrene. Bertrand extracted five maggots from the patient's ear, and by the following day all of the symptoms had disappeared.

Otalgia is certainly one of the most frequent reflex symptoms among those which are referred to the ear. All at once, without prodromes, a patient is suddenly seized with pains in the ear more or less severe and persistent; hot applications, ordinarily soothing, afford only a passing alleviation. Audition remains good; often there is no trace of irritation about the membrana tympani, inspection of the mouth shows one or more carious teeth on the side of the affected ear. The carious tooth may not even be sensitive; often, indeed, the patient is ignorant of the defective teeth. But in these cases extraction of the tooth instantly cures the otalgia.

Conversely, a disease of the ear may cause dental pains, without caries. I saw a patient [female] with reflex dental pains, associated with catarrhal otitis, who was anxious to have the non-carious tooth extracted. The dentist, not consenting, because the tooth was sound, thought the trouble originated in an empyema of the maxillary sinus, and was going to perforate the sinus, when the patient asked him to telephone me. Some explanations sufficed to establish the nature of the troubles and to prevent useless operations upon organs perfectly healthy. Two years before the same patient had had a similar attack of otitis with intense neuralgic pains, which were cured by a myringotomy; and the operation, repeated a second time in the same conditions, had the same satisfactory result.

Green reports a case of otalgia having coincided with the appearance of a cervical adenitis and disappearing with the latter. It is quite possible that in these cases a lesion of the nose or of the pharynx has caused the adenitis and otalgia. Certain diseases of the pharynx, among others ulcers upon the pillars of the palate, ulcerous pharyngitis, tonsillitis, lesions at the base of the tongue, sometimes produce violent pains in the ear, followed often by an inflammation of the tympanum. Conversely, a lesion of the middle ear may cause pains in the region of the larynx and provoke efforts of coughing.

Inflammation of the tympanic cavity, especially purulent otitis, may, by compressing or destroying the facial nerve, produce total or partial paralysis of it. Of all the affections of the middle ear, it is purulent otitis which produces most of the conditions of hyperemia, swelling and exudation favorable to injuring the facial nerve and interfering with its normal function. The aqueduct of Fallopius presents an opening above the fenestra ovalis, in its horizontal portion, which is constant in the new-born and very frequent in the adult. This anatomical arrangement explains the facility with which purulent inflammation is able to attack the facial. Thus it is not rare to observe a facial paralysis in the course of a suppurative otitis; more frequently a light paresis, followed by a sluggishness of the orbicularis and some lachrymation. As the facial has certain functions to perform in the middle ear, its alteration determines troubles which I will now rapidly review.

Hearing is increased or diminished. Deafness results from paralysis of the stapedius muscle innervated by the facial. The unopposed action of the tensor tympani crowds the base of the stirrup into the vestibule and probably compresses the labyrinth. Another symptom is tinnitus. Facial paralysis may cause also what is called the *paracusis of Willis*, an exaggeration of hearing. The mechanism of this paradoxical phenomenon is not well understood; but I will not stop to relate the different theories which have been adduced to explain it. The degree of the facial paralysis should be studied with care. Especially should one direct his attention to the vault of the palate; the participation of this part in the paralysis indicates that the nerve lesion has probably involved the geniculate ganglion.

Lesions of the chorda tympani or of the tympanic flexus produce symptoms which relate to the functions attributed to the facial, trigeminus, glossopharyngeal and sympathetic. As the branches of the trigeminus and glossopharyngeal contain tactile and gustatory filaments, diseases of the middle ear may produce neuroses of touch or taste; in the zone of the trigeminus, from the base to the point of the tongue, and in that of the glossopharyngeal, along the posterior third of the tongue, the vault of the palate and the posterior wall of the pharynx. When one is applying dressings in the canal in the treatment of purulent otitis with perforation, and touches the chorda tympani with the probe or some medicament, a sensation is perceived along the side of the tongue, which is variable, [being] tickling or salty, or a sensation of cold, or of taste, or of pain at the point of the tongue. Section of the chorda tympani causes a destruction or diminution of the sense of taste. Mechanical irritation of the wall of the tympanic cavity can produce an itchy sensation along the posterior pharyngeal wall. Inflammation [here] may produce the same feeling and cause the sensation of a foreign body in the pharynx and give rise to useless efforts at expulsion.

An anomaly of touch does not necessarily accompany an anomaly of taste; one may be lost, the other saved; their association only shows that the [nerve] lesion has involved both gustatory and tactile fibers. The reaction of diseases of the middle ear upon the salivary glands is evident, [as shown by the fact that] an abundant salivation may be induced by blowing powdered boracic acid in the auditory canal when the membrana tympani is perforated. This phenomenon finds its explanation in the fact that the nerves to the salivary glands contained in the branches of the facial and trigeminus proceed in great part from the facial; the submaxillary and sublingual glands receive their secretory fibers from the chorda tympani—the parotid, on the contrary, from the small superficial petrosal nerve to which filaments of the facial are carried by a branch anastomosing with the tympanic plexus. An irritation of the tympanic plexus in the middle ear may react, then, upon the submaxillary and parotid glands and cause an increased salivary secretion. Therefore it is important not to forget this reflex action in those subjects who complain of constant salivation, and who attribute this symptom to a disease of the throat or a nasal catarrh.

Besides the reflex nervous symptoms, which proceed from the immediate neighborhood of the ear, there are others which involve even the nervous centers, and which manifest themselves by alarming phenomena. I have seen cases of mania supervene in the course of diseases of the middle ear, purulent otitis or sclerosis, with intense and constant subjective noises. One patient told me that she continually heard the voice of her dead mother who said to her, night and day, *pray for me, pray for me.* These reflex psychoses, and those less grave which announce themselves by a changed disposition, a modification in the character, an intellectual sluggishness, with loss of memory, have been studied with care by Koeppe, who attributes them to an irritation of the trigeminus.

I will close this study by reporting three cases of remarkable reflex phenomena, caused by lesions of the ear. Some years ago a young girl consulted me in my office for deafness, which dated back several years. Examination revealed masses of cerumen. I commenced the injections and had completely cleansed one ear, when all at once, in injecting the other, my patient was seized with a grand hysterical attack, in which nothing was absent: passionate attitudes, circular and to-and-fro movements, etc. A little surprised at this unexpected complication, I remained a passive spectator of the scene, which renewed itself and threatened to be repeated indefinitely, when, recalling the experiences of Charcot at the Salpêtrière, I pressed firmly upon the ovaries aud restored the patient to her better senses. I ordered emollient instillations in the ear, and advised the patient to return another day. This time the attack of hysteria was not repeated. This case, although rare, is not surprising, if one remembers the ease with which an hysterical attack may be induced, especially in certain subjects.

The second case is that of S. A., a boy eleven years of age, who presented himself last May at the Notre-Dame Hospital in the department of nervous diseases for treatment of a hemichorea of the right side. The nervous trouble had existed almost three months, appearing in connection with an affection of the right ear. He was sent into my service for examination and treatment, and I found a chronic suppurative otitis media of the right ear. A discharge had existed for three months, since the hemichorea appeared, but the patient had had pains in the ear for nearly six months. After some weeks of treatment (carbolated glycerine, dry boric acid, etc.) the discharge ceased, but the nervous trouble still persisted when the patient quit my service. A little later, about a month after the cure of the ear, the hemichorea had improved; to-day the cure is almost complete.

A third case of reflex nervous trouble, emanating from the ear, was observed last summer. M. R., age twenty-three, weaver in a cotton factory, consulted me (August 11, 1896) at the request of a confrère of this city. She related that September 15, 1895, in stooping to take an object from the ground, she lost consciousness and remained in this condition for several hours. Upon recovery she had a violent cephalalgia, and was taken with vomiting, which threatened to recur every time she tried to raise her head. For some days which followed the accident this state persisted in all its intensity, and she lost consciousness for several minutes each day for three days afterward. The cephalalgia continued from the time of the accident, the patient finding no relief from the different treatments which she received; she was doctored and sat-up-with for many nights, and her death was even awaited at any moment. The disease continued in this manner almost a year, with all its original intensity, the temporary ameliorations not permitting the patient to resume her occupation. The doctors in charge of the case did not know what to think of it, so she was sent to Montreal to a confrère, and from him to me. Examination of her eyes revealed an astigmatism of one-half a diopter against the rule; nothing abnormal in the fundus of the eye; muscular functions normal; visual field and perception of colors intact; nothing to note in the nose or nasopharynx. In the left ear a mass of cerumen, deeply impacted, acting as a foreign body. The cerumen was removed, and, to the great surprise of the patient, she immediately experienced a relief, her head became clearer and the dizziness disappeared. For the first time she stated that she had not heard so well in the left ear as in the right. Questioned about this deafness, she stated that the noise in her head was such that she thought her brain was affected, and she had given little thought to her ear. Some days later the improvement was still more manifest. Cylindrical glasses were prescribed, to be worn constantly. Some months later the patient considered herself cured, although more subject than before the accident to light attacks of cephalalgia.

